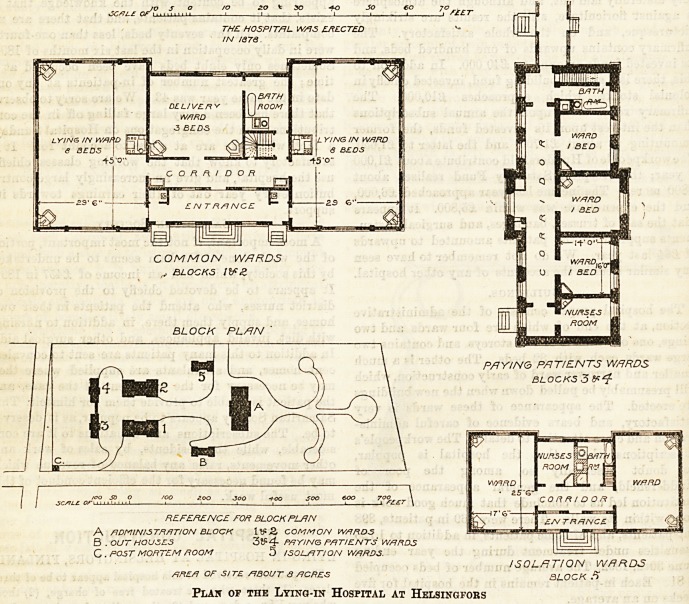# Lying-In Hospital at Helsingfors. Finland

**Published:** 1893-02-18

**Authors:** 


### HOSPITAL CONSTRUCTION.

### LYING-IN HOSPITAL AT HELSINGFORS, FINLAND.

The patients received at this hospital appear to be of three classes—(1) those who are treated free of charge, (2) those who pay 1 fm. a day, and (3) the well-to-do, who pay from 4 to 6 fm. a day.

The buildings are eight in number, and consist of: A, the administration block; B, outhouses; C, post-mortem room; I, ward block for Class 1; II., ward block for Class 2; III. and IV., ward blocks for Class 3; and V., "reserve" wing for isolation purposes.

All these blocks are entirely detached with the exception of III. and IV., which are connected together by a corridor.

Of the arrangements of the administration block, the outhouses, and the post-mortem room, the printed description gives no particulars.

Blocks I. and II. are alike in every particular, except that while the former is built of wood, the latter is built of brick. Each block contains a delivery-room with three beds, of which two only are usually occupied at one time. This room is about 24 feet by 22 feet, and about 14 feet 9 inches high. Thus each patient has over 2,500 cubic feet of air space. On one side of the delivery room is a ward for eight patients with their babies and two female students. This ward is rather more than twice the size of the delivery-room. On the other side of the delivery-room is a bath-room, which is also used as a ward kitchen, and a staircase. Adjoining these last is a similar ward to the other. The two large wards are connected together by a sort of double corridor, and each ward has a door opening on to the outer and one on to the inner corridor. In the outer corridor are two water-closets.

Block III. is built of brick, and contains three wards, usually occupied by one patient each, a nurses'-room, a bath-room, and a w.c. off the corridor. The nurses of Blocks I. and II. sleep in the corridor. Block IV. is similar in all respects, but is built of wood.

Block V. contains two wards, a nurses'-room, and a bath-room, with a double corridor similar to those in Blocks I. and II. These wards are intended for cases of puerperal fever, syphilitic patients, or those suspected of being infected in such a way as to be dangerous to others. The wards are warmed by open grates, in connection with which are ducts for fresh air, which pass under the floor from the outside wall to the sides of the grate, and thence discharge the warmed air at the upper part of the room.

It is curious to find in a country so far north as Finland a hospital of this nature with no covered communication whatever between the different blocks and the administrative building, when in this country it is still looked upon as a necessity to connect every part by closed-in passages. The whole idea of this hospital is, indeed, the very reverse of what would be thought necessary in a similar institution in England. The large area of ground occupied, the distance between the various blocks, and the one-storey blocks themselves would be an impossibility in a large English town. Impracticable as such a plan may be for an ordinary lying-in hospital here, does it not suggest a useful lesson in relation to the arrangement of the lying-in wards for workhouses? For it is to these, and not to the voluntary hospitals, that we must look in the future for perfection of arrangement for poor parturient women.

PLAN OF THE LYING-IN HOSPITAL AT HELSINGFORS

## Figures and Tables

**Figure f1:**